# Acute myocardial infarction following a Russell’s viper bite: a case report

**DOI:** 10.1186/1755-7682-6-7

**Published:** 2013-02-23

**Authors:** Mahboob Niraj, Jayamalee L Jayaweera, Indunil WGD Kumara, Nirmali WAJ Tissera

**Affiliations:** 1 National Hospital of Sri Lanka, Colombo, Sri Lanka

**Keywords:** Snake bite, Russell’s viper, Myocardial infarction, ST elevation myocardial infarction

## Abstract

**Introduction:**

Snake bite is a common and devastating environmental hazard, especially in rural areas of tropical countries. Acute myocardial infarction caused by snakebite has rarely been reported. To our knowledge we found only 10 cases of Myocardial infarction following a viper bite in English literature.

**Case presentation:**

We report a case of inferior ST elevation myocardial infarction following a Russell’s viper bite in a 37 year old healthy Sri Lankan (South Asian) female with no past history of cardiac disease or cardiac risk factors who died 30 hours following the bite.

**Conclusion:**

The course of events with respect to myocardium suggests a direct toxic effect of the venom on myocardial tissue or coronary vasoconstriction. Physicians should bear in mind the complications and devastating sequela of Myocardial infarction following Russell’s viper bite.

## Introduction

Sri Lanka has one of the highest snakebite rates in the world. Although there are 92 species of snakes found in the country, much of the morbidity and about 95% of the mortality associated with snakebites are due to the highly venomous cobra, Russell’s viper and kraits [1]. Local and systemic complications of snakebite have long been appreciated. Clinical picture following viper bite is usually characterized by local tissue reaction, haemorrhagic manifestations, nephrotoxicity and neurotoxicity in case of Russell’s viper. Myocardial involvement is rare with viper bites and Acute myocardial infarction (MI) caused by viper bite has been reported only a few times. The author is aware of only 10 cases of acute myocardial infarction complicating a viper bite in the English literature [2-11]. Most of these patients described were free of known cardiac disease or cardiac risk factors. Acute myocardial infarction was the primary problem in these patients. We describe herein a case of inferior myocardial infarction following a Russell’s viper bite.

## Case presentation

In September 2011, a previously healthy 37 year old Sri Lankan female with no history of cardiac disease or cardiac risk factors was brought to medical casualty ward within half an hour after a snake bite. The dead snake also was brought and identified as Russell’s viper (Daboia russelii). On admission patient was conscious and complained of pain at the bite site. On examination, fang marks were identified on the dorsal aspect of the 5th toe of left foot. Her heart rate was 80 beats per minute and blood pressure was 100/60 mm Hg, her temperature 37.2°C, and respiratory rate was 16 breaths per minute. Rest of the examination was unremarkable. The bedside test of 20 min Whole Blood Clotting time (20 min WBCT) was normal (<20 min). The baseline investigations of Full blood count haemoglobin (Hb) 11.8 g/dl, WBC 6200/mm3, neutrophils 55% lymphocytes 42% platelets 141000, Urine full report normal, Serum Na 135mmol/l, K 4.2 mmol/l and serum creatinine 0.9 mg/dl were normal. She was given acetaminophen, Intravenous (IV) Antibiotics, IV Normal saline slow drip and Tetanus toxoid. The 20 min WBCT was repeated half an hour later was prolonged. In view of systemic envenoming, Indian polyvalent snake anti venom serum (AVS) was planned to be given. While awaiting AVS treatment she developed retrosternal tightening chest pain, faintness, and vomiting. The patient was profusely sweating with thready pulse of 114/min, systolic blood pressure (BP) was barely recordable at 50 mmHg and tachypnoeic, with respiratory rate of 36/min. On Auscultation, lungs had bi basal crepitations and patient was in cardiogenic shock. Electrocardiogram showed acute ST elevations on inferior leads (lead II, III, aVF) with sinus tachycardia. Despite IV fluids she had low BP. She was started on IV dopamine infusion and AVS was given, but thrombolysis, anticoagulants and antiplatelets were not considered due to prolonged 20 min WBCT. By this time patient had excessive puncture site bleeding but no other bleeding manifestation.

Coagulation profile which was done 4 hours after the Myocardial infarction revealed prolonged prothrombin time (PT) to 31.8 seconds (control 12 sec). Activated Partial Thromboplastin Time (APTT) was >180 s (control 25–40 sec). Cardiac enzyme Troponin I was elevated. Since patient was developing acute pulmonary oedema with dropping arterial Oxygen saturation (SpO2), she was transferred to an Intensive care unit and assisted ventilation was given. Due to frequent hypotension and prolonged 20 min WBCT AVS therapy was repeated. Her Blood pressure was dropping frequently but responded to Subcutaneous Adrenaline boluses and IV fluid boluses and IV inotropes were stepped up. Patient’s urine output was >0.5 ml/kg/hr. Chest X ray showed evidence of pulmonary oedema. Facilities for point of care Echocardiogram was not available.

Repeat Laboratory investigations showed a Hb of 14.9 g/dL, white blood cell count 33700/mm3 with 96% neutrophils and 4% lymphocytes. The platelet count was 117000/mm3 and Serum creatinine 1.7 mg/dl, Serum electrolytes and Random plasma glucose levels were within normal limits. Liver enzymes SGOT 316 u/l, SGPT 311 u/l. D-Dimer 3.2 mg/l (<0.2 mg/l). 30 hours after admission the patient’s BP became further unstable despite AVS, Inotropic support and IV fluids. Half an hour later asystole occurred, and cardiopulmonary resuscitation was not successful.

## Discussion

The exact mechanism by which snake bite envenoming leads to myocardial infarction is unclear. Various mechanisms that have been suggested as causative for myocardial infarction following viper bite, are as follows: - 1. Hypovolemic shock due to bleeding (bleeding due to hemorrhagins or toxic vasculitis). 2. Anaphylactic shock. 3. Hypercoagulability in consumption coagulopathy [8]. 4. hyperviscosity secondary to hypovolemia induced haemoconcentration. 5. Direct cardiotoxic effect on myocardium [3,5]. 6. Coronary spasms [4,10] due to endothelins or sarafotoxins [12] or anxiety.

Dissanayake P *et al.*[6] have described acute MI following Russell’s viper bite in a 47 year old man and possibility of predominant coagulant in venom resulting in coronary thrombosis and anaphylactic shock leading to MI were discussed as causes.

In the timeline of events it is noted that retrosternal chest pain followed by cardiovascular collapse 1 hour after the bite is suggestive of coronary spasm as an important possible etiological factor for MI in our patient. The direct cardiotoxic effect of snake venom can result in myocarditis and extensive myocardial necrosis. It has been reported in two horses injected with Viper-palaestinae venom for commercial production of antibodies [13]. This possibility is less likely in our case because myocarditis damages myocardium diffusely and in our patient, the ST segment elevations were limited to inferior leads only. Hypercoagulopathy due to consumption coagulopathy causing thrombus in coronary vessel is also a possibility in this case.

## Conclusion

This case report describes a fatal outcome in a 37 year old female caused by a Russell’s viper bite as a result of combined effects of cardiogenic shock due to myocardial infarction, pulmonary oedema, consumption coagulopathy and renal impairment. Although myocardial damage does not seem to be a common feature of Sri Lankan Russell’s viper bite, Physicians should bear in mind, the complications and devastating sequela of Myocardial infarction following Russell’s viper bite.

### Consent

Written informed consent was obtained from the patient’s next of kin for publication of this case report. A copy of the written consent is available for review by the Editor-in-Chief of this journal.

## Competing interests

The authors declare that they have no competing interests.

## Authors’ contributions

MN and LJJ were involved in literature search, interpretation of the case and drafted the manuscript. Both MN and LJJ have equally contributed to this manuscript. WGIDK helped substantially in literature search and drafting the manuscript. WAJNT did the critical revision for important intellectual content in the manuscript and gave the final approval of the version to be published. All authors read and approved the final manuscript.
